# Effect of Elevated Temperature and Annealing Time on Mechanical Properties of Ti/Cu Bimetal

**DOI:** 10.3390/ma15238707

**Published:** 2022-12-06

**Authors:** Robert Uścinowicz

**Affiliations:** Department of Mechanics and Applied Computer Science, Faculty of Mechanical Engineering, Bialystok University of Technology, 45C Wiejska, 15-351 Bialystok, Poland; r.uscinowicz@pb.edu.pl

**Keywords:** metallic layered structure, tensile test, mechanical properties, hardening, micrography

## Abstract

The paper presents the results of the elastoplastic properties of Ti/Cu bimetallic rods. They were obtained by extrusion and composed of a copper core with a covered titanium layer. Experiments were carried out at room temperature on virgin samples, and samples were subjected to prior annealing in the temperature range of 600–900 °C for 30, 60, and 90 min. The modern technique of impulse excitation of vibration was used to analyze the elastic properties of bimetal, obtaining the temperature and time characteristics of Young’s modulus, internal friction, and resonance frequency variability. Subsequently, the samples were stretched to breakage, obtaining information on the values of limit stresses, their deformability, and the energy demand for uniform elastic–plastic deformation in terms of the effect of temperature and annealing time. The influence of thermal processes on the strengthening of the Ti/Cu bimetal was also examined, and microscopic observations and qualitative analysis of the diffusion zone at the interface of the phases were carried out. The research was to answer the question of how a short-term temperature increase in 600–900 °C affects the physical properties of Ti/Cu bimetallic rods. These rods were used as a high-density electric current carrier in metallurgical processes in environments of aggressive chemical compounds. Studies have shown that short-term annealing at elevated temperatures causes a drastic reduction in the strength of the Ti/Cu bimetal, leading to structural changes within the components, and the diffusion zone with the release of intermetallic compounds, leading to structural degradation. Heating at 900 °C for 60 and 90 min caused accelerated interface degradation and destruction of the Ti/Cu bimetal by delamination.

## 1. Introduction

The energy and raw material crisis that has occurred in recent years has triggered the need to reduce the energy consumption of the mining and processing industries as well as the demand for expensive metals and minerals. Metal sandwich composites, which contain already known metals and metal alloys in their structure, are the answer to these challenges. By undertaking their production, practically new metal materials with multifunctional physical properties are obtained, in which each of the layers plays a different role in the structure. The technological development of methods of joining metal layers by explosive welding, extrusion, hot and cold rolling, cladding, chemical deposition or gluing allows for obtaining a metal structure with high strength. Additionally, a more functional, economically advantageous material with intelligently designed physical properties is obtained [1].

The layered metal composite composed of copper and titanium in the form of a rod or a pipe is widely used in the electrochemical industry. The copper layer is a very good conductor of electricity, and titanium has high corrosion resistance to many aggressive chemical compounds, especially in acid and chloride environments [2]. Very often, rods obtained from joining these metals are used as anodes in electrolysis processes [3,4,5]. They also play the role of cathodes in many electrochemical processes [6]. Occasionally, as shown by Du et al. [7], in such a copper/titanium galvanic pair left in seawater, copper pitting corrosion may occur due to potential differences between the components, especially when the copper is pure.

Another application of the Ti/Cu composite are electrical busbars bonded by a mechanical or metallurgical method, which is used to ensure an even distribution of the flowing electric current, ensuring the extreme strength of the structure at thermal cycles [8]. At the same time, these bimetals ensure high corrosion resistance and can be easily shaped and welded. Shmorgun et al. [9] also mentioned the use of a composite based on titanium and copper in cryogenic and exchanger devices for thermal barriers to heat protection. Ti/Cu bimetal is also used for abrasion-resistant intermetallic coatings [10,11] and heat-resistant elements [12].

Information on the study of the mechanical properties of Ti/Cu bimetal at room temperatures using tensile tests can be found in the paper of Zhu et al. [13]. Tests were an introduction to the 3D modelling of the rotary Ti/Cu tube bending process. Shear tests of bimetal samples in the form of a single lap joint, obtained by explosive welding of copper and titanium plates, were carried out by Kahraman and Gulenc [14]. The researchers found that the bimetal interface strength was higher than that of the copper plate and that cracking occurred within the copper plate, while the Scanning Electron Microscope (SEM) tests showed no traces of intermetallic compounds at the interface. Similar shear tests of bimetallic Ti/Cu samples obtained from explosive bonding were performed by Paul et al. [15]. The research work contained an extensive analysis of the microstructure of the layer connection zone and focused on the parameters of the explosive bonding process.

The secrets of the process of hydrostatic extrusion of bimetal Ti/Cu alloy, where the technique of visco-plastic pressing of a pressure medium was used, are described in the paper of Matsushita et al. [16]. The authors observed the formation of intermetallic compounds at the interface between titanium and copper alloy when the extrusion and annealing temperature after extrusion was high. The shear bond strength, determined in tests, was as high as 120–150 MPa, which qualified them for applications for titanium-plated copper electrodes. In the paper of Lee et al. [17], the Ti/Cu bimetal obtained by indirect extrusion was characterized. It has been noticed that the interfacial layer composed of hard intermetallic phases, depending on its thickness, may reduce the bond strength.

There are some research works in the literature on Ti/Cu bimetal obtained by explosive welding. Paul et al., in their papers [18,19], devoted much attention to the study of the bond zone of metal layers and the influence of thermal processes on the Ti/Cu sheet. They analyzed the influence of annealing at 700 °C on the interface microstructure and also discussed the effects of recrystallization. The authors also identified the metallic phases which are contributed by the influence of heat. They also found that a large drop in strength, as evidenced by the tensile-shear test, was observed in the annealed specimens after only 0.25 h compared to the virgin material. In another paper, Zu et al. [20] analyzed the processes taking place in the Ti/Cu joint obtained by explosive welding as a process of interfacial deformation and thermal diffusion. The authors observed the annealing process in terms of microstructure changes and observed the brittle fracture effect of the interface. They noticed that in the interfacial bonding mode, the shear strength of the bimetal was 140 MPa. Pronicev et al. [12] carried out studies of the Ti/Cu bimetal by testing the thermal conductivity of the composite and the phases formed at the interface of the joint. They assessed the thickness of the diffusion zones after long heat-exposure of 30 and 60 h and identified metallic phases. Executive parameters of Ti/Cu explosive bonding were the subject of interest of Możejko et al. in the paper [21].

Diffusion bonding of copper and titanium or titanium alloy was considered in the paper of Aydin et al. [22]. This technique was adopted by trying to combine Ti and Cu metals under a stress pressure of 3 MPa at the temperatures of 875, 890 and 900 °C for 15, 30 and 60 min. The quality of the joint was assessed by measuring the microhardness and the shear test. The identification and analysis of interfacial relationships in the transition zone were also performed, and the depth of diffusion of both metals was determined, which was dependent on the temperature.

The subject of interest of Hosseini et al. [23,24] was a bimetallic nano-structured composite based on thin copper and titanium layers obtained by accumulative rolling-bonding (ARB). For the material thus produced, a detailed observation was carried out using electron microscopy and X-ray spectrography, showing the formation of titanium reinforcements in the fine-grained copper structure and shear bands resulting from crushing. In another paper [25] by these authors, tests of the three-layer Ti/Cu/Ti composite obtained by the rolling method were carried out, looking for optimal conditions for joining components. The peel test was used to assess the strength of the structures.

The tests were conducted on Ti/Cu rods, which are widely used in the electrotechnical, metallurgical, and chemical industries. They have found applications as new materials in electrotechnical installations, electrolysis, electroplating processes, and metallurgy. The bars’ manufacturing process requires high plastic deformation, which causes internal stresses, and deforms crystalline texture, so heat treatment is required to eliminate these adverse effects. Incorrect selection of its parameters (temperature, time of thermal exposure) may change the mechanical properties of the bars. Correcting these parameters is difficult due to the bimetal structure, i.e., the presence of physically different metals.

On the other hand, in operating conditions, the transmission of high-intensity electric current through the electrical installation made of Ti/Cu bimetal creates the risk of overheating, which may result in the loss of its original favorable mechanical properties, including strength.

The research aimed to respond to the threats caused by the above factors. Research activities were connected to analyzing the effect of heat in the temperature range 600–900 °C and variable holding time on selected mechanical properties of the Ti/Cu bimetal, i.e., internal friction parameter, specific energy of uniform elastic and plastic strain, limit stresses, including the strain hardening process. The original impulse-excitation technic method was used concerning the inhomogeneous material to evaluate the elastic properties of the tested bimetal. The work also analyzed the fractures of the samples with the microscopic assessment of the connection quality at the layer separation zone.

## 2. Experimental Procedure

The experimental studies were conducted in four stages. The first stage involved carrying out tensile tests at room temperature on thin-walled bimetallic Ti/Cu tubular samples and on samples without a titanium or copper layer, i.e., Ti/Cu bimetal components. The aim of these studies was to understand the mechanical properties of the Ti/Cu bimetal components. The results of these tests are described in chapter 3 of the paper [26]. In the second stage, the elastic properties of the Ti/Cu bimetal were investigated using the acoustic resonance method. In the third stage, tensile tests (essential) were carried out on properly prepared Ti/Cu bimetallic samples. The last stage (fourth) was to assess the microscopic quality of the bonding of titanium and copper layers after thermal interactions. The samples for the tests in the second, third, and fourth stages were heated at the temperature of 600–900 °C for 30–90 min.

### Materials and Specimens

The starting material for the tests were Ti/Cu bimetallic bars with a nominal diameter of *ϕ* 12 mm and a length of 6 m. Each bar consisted of a copper core with a diameter of *ϕ* 8.8 mm and a titanium coating 1.6 mm thick distributed concentrically along the circumference of the core. The bimetallic rod was made by extrusion with a permanent connection of metals without a transition layer. As reported in the literature [27], the Ti/Cu rods are most often hydrostatically extruded at the temperature of 650–700 °C, which allows for obtaining the shear strength of the layers in the order of 120–150 MPa. The chemical composition of the tested Ti/Cu bimetal components was determined by the manufacturer [28] and is presented in Table 1. The mean volumetric shares of the components were: $f_{Ti}=0.46$, $f_{Cu}=0.54$.

**Figure 1:**
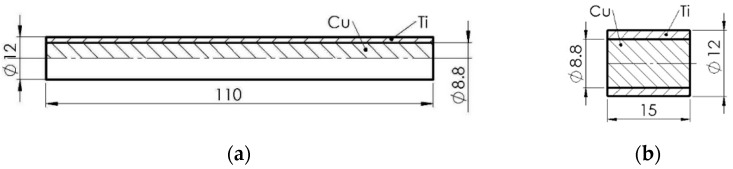
The geometry of the samples used in the tests: (**a**) sample for the tensile test, (**b**) sample for microscopic analysis.

The initial form for making the samples was bimetallic bars with a nominal diameter of *ϕ* 12 mm and a length of 125 mm. The geometric axis of the samples was in the direction of extrusion. These samples were divided into two groups. The first group consisted of samples without thermal treatment (indication-*T* = 20 °C). The second group of samples was annealed at the following temperatures: 600, 700, 800, and 900 °C, respectively, and independently maintained at these temperatures for 30, 60, and 90 min, after which they were all cooled in the air. After these treatments, each sample from both groups was cut into two sections. The longer part (Figure 1a), with a nominal diameter of *ϕ* 12 mm and a length of 110 mm, was a sample for the evaluation of elastic properties using the IET (Impulse Excitation Technique) and uniaxial quasi-static tensile tests. The shorter sample (Figure 1b) was used for SEM (Scanning Electron Microscope) microscopic analyses. All mechanical tests were carried out at room temperature (20 °C).

## 3. Elastic Properties of Ti/Cu Bimetal after Thermal Action

The dynamic method described in the ASTM E1876-15 standard (American Society for Testing and Materials) [29] was used to measure the elastic properties of bimetal due to the shape and dimensions of the samples. An acoustic frequency resonance analyser (Resonance Frequency Analyzer Device (RFDA)), IMCE NV, Genk, Belgium was used, and the same samples (Figure 1a) were used in the uniaxial tensile tests. With the help of this technique, Young’s modulus *E* (in the direction perpendicular to stratification), basic resonance frequencies *χ_f_*, and internal friction parameters *Q*^−1^ were determined.

The applied method used the phenomenon of acoustic resonance, i.e., the increase in the vibration amplitude of the three-point (elastically) bending sample when the frequency of external forcing vibrations was close to the natural frequency of its material. It allowed for obtaining both quantitative information on the value of its modulus of elasticity as well as qualitative information on its integrity [30]. An indispensable phenomenon occurring here was the simultaneous damping, which caused the reduction in the amplitude of free vibrations in the exciting vibrating system, as a result of which elastic energy was dispersed.

The performed measurement with the dynamic method consisted in inducing a mechanical impulse (impact with an impulser) and causing a mechanical wave (vibrations) in the tested sample subjected to three-point bending. The resulting vibrations had a frequency spectrum consistent with the resonance frequency of the Ti/Cu bimetallic sample. With the use of an acoustic transducer (microphone), analogue electrical signals were sent to a computer and further, using mathematical algorithms and software, the vibration spectrum was analyzed, and the signal parameters were calculated. The RFDA measuring system from the IMCE Company was used. With the knowledge of the mass and geometry of the sample, it was possible to determine its elastic properties and acoustic parameters. Detailed information on this measurement technique can be found in Roebben et al. [30,31]. The analysis of errors in this method was discussed in the paper of Raggio et al. [32]. This method has been successfully applied to determine the elasticity constants of structurally complex materials by Song et al. [33] and by Uscinowicz [34].

For the cylindrical Ti/Cu bimetallic samples in the three-point bending mode, Young’s modulus was calculated from the following formula [35]:

(1) $$E=1.6067\cdot \left(m\cdot \chi_{f}^{2}\right)\cdot \left(\frac{L^{3}}{D^{4}}\right)\cdot S\;,$$

where *E*—Young’s modulus, *m*—sample mass, *L*, *D*—length and diameter of a cylindrical sample, respectively; *χ_f_*—basic resonance frequency during bending of the sample; *S*—correction factor for calculations taking into account the finite bar thickness, Poisson’s ratio, etc. [35].

The measurements carried out with the dynamic method described above allowed us to determine the significant parameter, which was the internal friction *Q*^−1^, which was defined by the following relationship:

(2) $$Q^{-1}=\frac{\Delta W}{2\pi W}\;,$$

where *W*, Δ*W*, respectively, accumulated and lost energy per volume unit of a vibrating solid during one period.

Fourier analysis was used to calculate the internal friction parameter *Q*^−1^, and it was calculated from the formula:

(3) $$Q^{-1}=\frac{k}{\pi \chi_{f}},$$

where *k*—equation parameter, the exponential decay parameter of the vibration component.

The values of the resonance frequencies *χ_f_* were also determined, which occurred in the vibration equation *x*(*tp*):

(4) $$x\left(t\right)=Ae^{-kt_{o}}\sin(2\pi \chi_{f}t_{o}+\varphi ),$$

where *A*, *φ*—parameters of Equation (4); *χ_f_*—resonant frequency in the three-point bending mode; *t_0_*—time parameter.

### Results of Impulse Excitation Tests and Discussion

**Figure 2:**
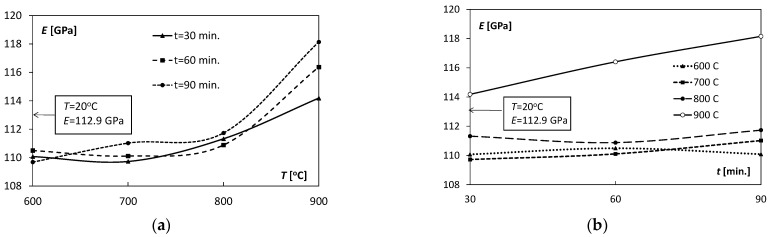
Variations of values of Young’s modulus *E* of the Ti/Cu bimetal dependent on: (**a**) temperature *T*, (**b**) annealing time *t*.

**Figure 3:**
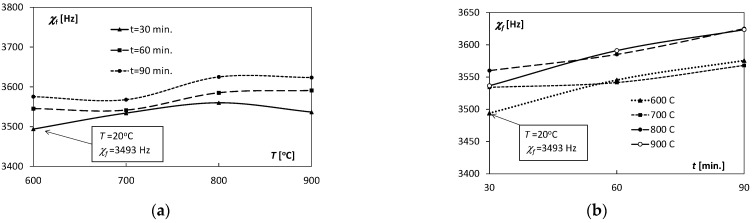
Dependence of the internal friction parameter *χ_f_* on: (**a**) temperature *T*, (**b**) annealing time *t*.

**Figure 4:**
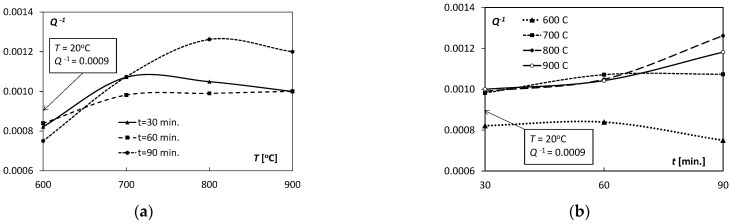
Dependence of the internal friction parameter *Q*^−1^ on: (**a**) temperature *T*, (**b**) annealing time *t*.

The values of the elastic properties of the Ti/Cu bimetal obtained from the acoustic resonance tests are presented in Table 2, and their variability depending on the temperature and the annealing time are shown in Figure 2, Figure 3 and Figure 4.

With the increase in temperature, the values of Young’s modulus *E* in the temperature range of 600–800 °C when the samples were soaked for 30, 60, and 90 min were similar and oscillated around the value of 110–111 GPa (Figure 2a). A significant increase in the value of these modules to the level of 114.2 GPa for 30 min and 116.4 GPa for 60 min and 118.1 GPa for 90 min, respectively, was observed for the annealing temperature of 900 °C. In this case, it was the result of high temperature and structural changes in both the titanium and copper layers due to the formation of brittle intermetallic phases, which was also found in the paper [22]. An increase in the holding time in the range of 30–90 min (Figure 2b) did not cause significant changes in the modulus values *E* for the samples to withstand temperatures of 600–800 °C. In the case of samples heated to the temperature of 900 °C, a strong linear increase in the value of *E* modules was observed with the holding time *t*. The average value of Young’s modulus *E* of the unannealed samples (*T* = 20 °C) was *E* = 112.9 GPa and was significantly higher than for the samples subjected to heat treatment, but with the exception of the annealing temperature of 900 °C. It follows that Young’s modulus is not a physical constant but a property that is influenced by many factors. As Puskar [36] argued, the definition of the ideal Young’s modulus can only be used for isotropic, single-phase and defect-free metals deformed within the elastic stress range.

The variability of the measured values of the resonance frequencies $\chi_{f}$ of the Ti/Cu samples was also analyzed, the values of which depended on the temperature and time of heat exposure (Figure 3 and Figure 4). The values of the acoustic frequencies $\chi_{f}$ during the measurements ranged from 3493 to 3625 Hz. They were an integrity indicator of the connection of Cu and Ti layers as well as the source of information about reference values of the elastic reaction of the cylindrical sample to the mechanical excitation of vibrations. Curves of variation $\chi_{f}\left(T\right)$ (Figure 3a) were prepared based on measurements for samples annealed in the temperature range 600–900 °C for *t* = 30 min and *t* = 60 min They had a similar course, which proves a similar reaction of the material to a mechanical dynamic impulse. Figure 3b shows that the resonance frequencies $\chi_{f}$ with the increase in the annealing time, for all temperatures in the range 600–900 °C, increased quasi-linearly.

The parameter *Q*^−1^, defined by Equations (2) and (3), was an indicator of changes taking place in the Ti/Cu bimetal structure. The values of the parameter *Q*^−1^ for the holding time *t* = 30 and 60 min reached the maximum at 700 °C; for time *t* = 90 min, the maximum of *Q*^−1^ was at a temperature of 800 °C, and all the courses were strongly non-linear (Figure 4a). For bimetallic samples holding for 90 min at *T* = 800 °C, the value of the *Q*^−1^ parameter was 1.26 × 10^−3^, highest value observed in the tests. Figure 4b illustrates the variability of the parameters *Q*^−1^ with the increasing of holding time *t*. Characteristics *Q*^−1^(*t*) for samples annealed at *T* = 600 °C for 30–90 min significantly deviated from the other curves, taking the lowest test values. The curves *Q*^−1^(*t*) for *T* = 700, 800 and 900 °C had similar values for *t* = 30 and 60 min. The increase in the value of *Q*^−1^ with the temperature increase can be attributed to many factors that apply independently to both copper and titanium or the metallic phases at the interface appearing due to diffusion and the discontinuities emerging at the bonding interface of Ti and Cu. The qualitative analysis and review of the mechanisms causing these effects in the microstructure of tested pure metals can be found in the research work of Blanter et al. [37]. In his book [36], Puskar distinguishes three stages of the dependence of *Q*^−1^ on temperature, of which he treats the temperature 0.5–0.6 *T*_m_ (*T*_m_—melting point) as the limit for which there are linear dependencies. In the case of the tested Ti/Cu bimetal, it was more complex, as many effects often overlapped, worked together, or against each other, which made it difficult to isolate any particular mechanism assuming the factors mentioned above as probable.

## 4. Tensile Tests of Specimens after Thermal Action

As mentioned, the tensile tests were carried out on the same Ti/Cu bimetallic samples (Figure 1a) described above. Tensile tests were carried out according to the recommendations in the book of Davis [38] and the technical standard ASTM E8/E8M-11 [39]. The samples were loaded with speed $\dot{\varepsilon }=2\cdot 10^{-3}s^{-1}$ on the MTS 809.10 dynamic machine (MTS Systems Corporation, Eden Prairie, USA ). The deformation measurements were carried out using the Aramis 3D 4M digital image correlation system ( (Carl Zeiss GOM Metrology GmbH, Braunschweig, Germany). The tests provided basic information on the elastic–plastic properties of the bimetal under monotonic quasi-static loading, i.e., the limit stresses *R_p_*_0.05_ and *R_p_*_0.2_ corresponding to permanent (non-proportional) strains of 0.05% and 0.2%, tensile strength *R_m_*, and the uniform permanent strain $\varepsilon_{u_{max}}$. In addition, the values of the uniform plastic strain energy per volume unit *L_p_* and elastic *L_e_* were also determined. The obtained values are presented in Table 3.

**Figure 5:**
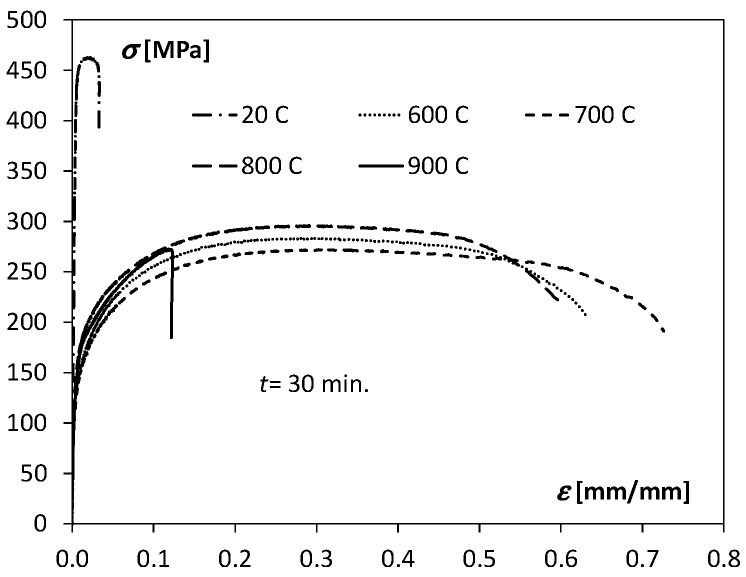
Exemplary of tensile curves for Ti/Cu bimetal samples unheated and annealed in the temperature range of 600–900 °C for *t* = 30 min.

Examples of tensile curves for unannealed and annealed samples in the temperature range of 600–900 °C for *t* = 30 min are shown in Figure 5.

**Figure 6:**
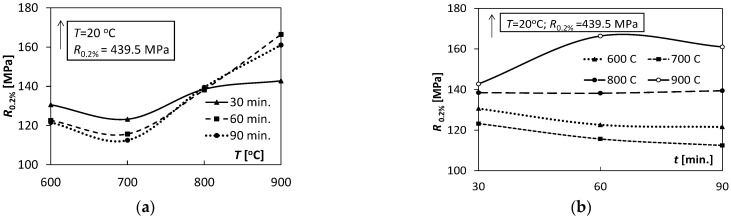
Variability of the value yield point *R_p_*_0.2_ depending on: (**a**) temperature *T*, (**b**) annealing time *t*.

**Figure 7:**
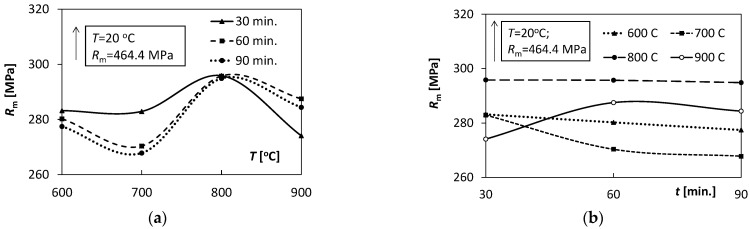
Dependence of the tensile strength *R_m_* on: (**a**) temperature *T*, (**b**) annealing time *t*.

**Figure 8:**
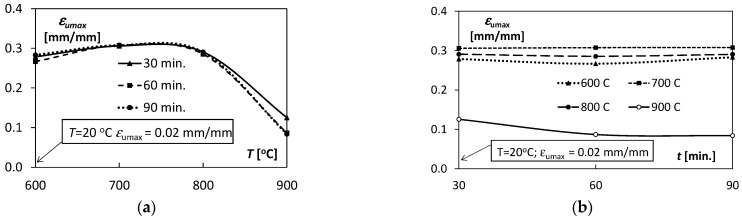
Dependence of the uniform plastic deformation value $\varepsilon_{u_{max}}$ on: (**a**) temperature *T*, (**b**) annealing time *t*.

**Figure 9:**
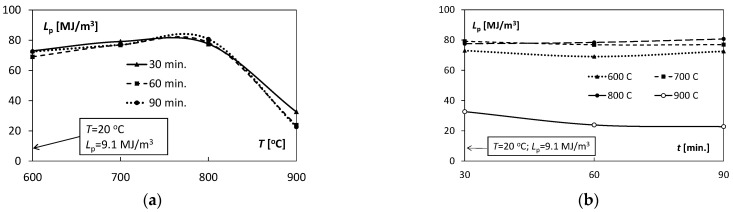
Variability of the uniform plastic strain energy per unit volume *L_p_* depending on: (**a**) temperature *T*, (**b**) annealing time *t*.

To better illustrate the influence of temperature and annealing time on the Ti/Cu bimetal mechanical properties, Figure 6, Figure 7, Figure 8 and Figure 9 were used.

### Results of Tensile Tests and Discussion

Comparing the tensile test results for all samples made of Ti/Cu bimetal, it can be concluded that the values of the mechanical properties for the samples without thermal effects (material as delivered) differed significantly compared to those for the samples subjected to heat (Table 3, Figure 6, Figure 7, Figure 8 and Figure 9). For example, the percentage relative differences for the ultimate stresses $R_{p0.05},$ *R_p_*_0.2_ and *R_m_* and the maximum stress values obtained for the samples after thermal action were: 65.3%, 63.4%, and 36.5%, respectively. Relative uniform plastic strain $\varepsilon_{u_{max}}$ for unannealed samples was 0.02, while the maximum after annealing was 0.31. The specific unit energy required for uniform plastic deformation of the virgin specimen *T* = 20 °C was about 71.6 MJ/m^3^ lower than the maximum values for the annealed specimens. Thus, annealing of the samples before the tensile tests resulted in a significant reduction in yield strength, strength, and at the same time an increase in the deformability of the samples, which could be expected.

With increasing the annealing temperature, the yield point *R_p_*_0.2_ reached the minimum value at the temperature of 700 °C for the holding times of 30, 60 and 90 min (Figure 6a), and the maximum value for T = 900 °C. A similar trend in maintaining the characteristics in the range *T* = 600–800 °C can be observed for the tensile strength values *R_m_(T)* (Figure 7a). However, in the case of the latter for 900 °C, there were significant decreases, which can be attributed to the effects of structural changes and energy exhaustion in the process of hardening of bimetal components. This effect distinguishes them from the observed characteristics in Figure 6a.

Figure 6b illustrates the change in value of *R_p_*_0.2_ with increasing annealing time. For *T* = 600 and 700 °C, there is a slight decrease in the value of *R_p_*_0.2_. For *T* = 800 °C, the values of *R_p_*_0.2_ were independent of the holding time. Strong non-linearity and large variability of values of *R_p_*_0.2_ are observed for *T* = 900 °C with the extension of the holding time from 30 to 90 min. On the other hand, Figure 7b shows a large variation in the tensile strength *R_m_* values for *T* = 900 °C with an increase in the holding time, and the stability of the values for *T* = 800 °C.

The above-mentioned uniform plastic strains, $\varepsilon_{u_{max}}$, for different annealing times *t*, characterized by a significant similarity of the values (Figure 8a) with the increase in annealing temperature *T*. Initially, the values $\varepsilon_{u_{max}}$ increased from 0.28 to 0.31 for 600–700 °C, and then finally decreased to the level of 0.08–0.13 for *T* = 900 °C. A significant similarity of the shape of the characteristics *L_p_* (*T*) illustrating the changes in the values of uniform plastic strain energy per volume unit (Figure 9a) in relation to the above-described characteristics $\varepsilon_{u_{max}}\left(T\right)$ (Figure 8a) was also observed. It proves the relation of the plastic uniform strain with the demand for indispensable energy to uniform plastic deformation.

With the increase in the annealing time t, all the characteristics $\varepsilon_{u_{max}}\left(t\right)$ illustrating the changes of $\varepsilon_{u_{max}}$ were almost linear. They exposed constant values independent of the holding time *t* (Figure 8b). A slight non-linearity of the characteristics was observed for T = 900 °C. Similar qualitative trends were for the characteristics *L_p_* (*t*) (Figure 9b) with increasing holding time *t* in the range of 30–90 min as for $\varepsilon_{u_{max}}\left(t\right)$ (Figure 8b).

## 5. Hardening of Ti/Cu Bimetal

The subject of the study was also the hardening process of the Ti/Cu bimetal during the monotonic tensile of non-heated and thermally influenced samples. Therefore, the test data were used for a mathematical description of the hardening process. The Swift equation [40] was applied here, using its three-parameter form:

(5) $$\sigma_{t}=K{\left(\varepsilon_{o}+\varepsilon_{t}\right)}^{n},$$

where, $\sigma_{t}$, $\varepsilon_{t}$—true stress and strain, respectively; $K,n,\varepsilon_{o}$—hardening curve coefficients; *n*—hardening coefficient.

**Figure 10:**
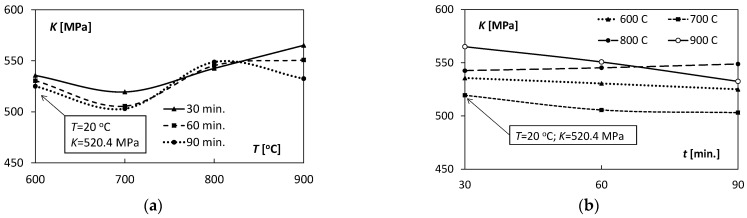
Dependence of the hardening coefficient *K* on: (**a**) annealing temperature *T*, (**b**) holding time *t*.

**Figure 11:**
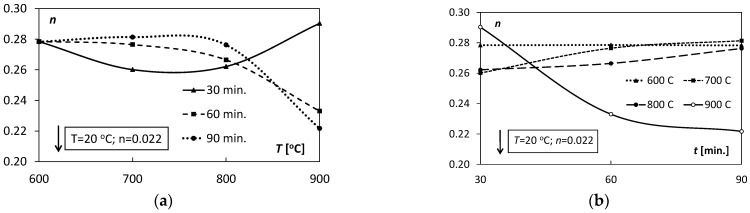
Dependence of the hardening coefficient *n* on: (**a**) annealing temperature *T*, (**b**) holding time *t*.

The coefficients *K, n* and $\varepsilon_{o}$ appearing in Equation (5) characterized the degree of deformation hardening of the Ti/Cu bimetal. The coefficients include both the effects of the hardening process during extrusion and the weakening processes resulting from the action of heat on the samples, i.e., heating and holding in the tested temperature ranges. The values of the above coefficients are presented in Table 4. The variability characteristics of the *K, n* coefficients are illustrated in Figure 10 and Figure 11.

The values of the coefficient *K* of the hardening curve, occurring in Equation (5), for the unannealed samples, were similar to the values of the analogous coefficients of the heat-affected samples, while the hardening coefficient *n* was over ten times lower than the coefficients for the annealed samples. The average values of the $\varepsilon_{o}$ coefficient for the unannealed samples suggest the semi-hard state of the bimetallic bar, pre-hardened. The positive values for the samples subjected to the thermal factor indicate an elongated segment of plasticity, which can be seen in Figure 5.

The analysis of Figure 6a and Figure 10a shows a substantial shape similarity of the *R_p_*_0.2_ (*T*) and *K*(*T*) characteristics for 30 and 60 min in the temperature range of 600–800 °C, which suggests a close relationship between the yield point *R_p_*_0.02_ and the *K* coefficient. However, in the case of the temperature *T* = 900 °C, these relations were inconsistent. With the increase in the holding time (Figure 10b), the values of the *K* coefficient decreased almost linearly in the case of *T* = 600, 700, 900 °C; for *T* = 800 °C they were almost unchanged.

Characteristics of the hardening factor *n*(*T*), depending on the increasing temperature *T* for 30–60 min range, were highly non-linear (Figure 11a). Characteristic *n*(*T*) for *t* = 30 min had a parabolic course with the minimum of the coefficient value of 0.26. For *t* = 60 min and *t* = 90 min values of this coefficient at the temperature range 600–800 °C oscillated around 0.27 and then decreased to 0.22 for *T* = 900 °C. The analysis of the diagram in Figure 11b allows evaluation of the influence of the holding time length on the values of the hardening coefficient *n*. For *T* = 600, 700, 800 °C, there was a slight linear increase in the coefficient value. However, in the case of T = 900 °C, a significant non-linear decrease in the values of *n* from 0.29 to 0.22 was observed. It proves a significant influence of the holding time on the hardening process at this temperature.

## 6. SEM Assessment of Ti–Cu Interface

**Figure 12:**
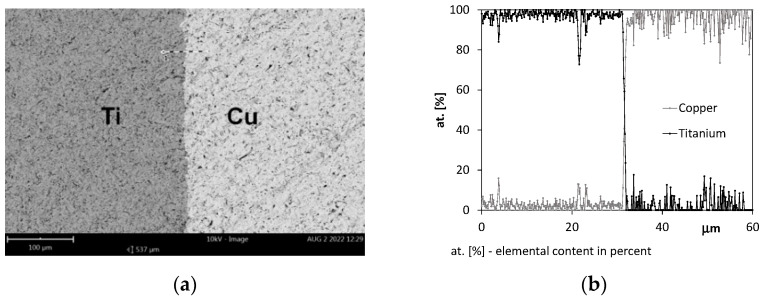
Contact zone of titanium and copper in the bimetal before the annealing process (*T* = 20 °C): (**a**) SEM photo, ×500, (**b**) linear analysis of Ti and Cu distribution in the contact zone.

The permanent bond of metal layers in the Ti/Cu bimetal plays a significant role in the operation of the entire bar structure A Phenom XL electron microscope performed the linear analysis of the interface zone between titanium and copper in the sample (in the condition as delivered). Figure 12 shows an image of the interface from an electron microscope and the line analysis in the contact zone of the titanium and copper layers (*T* = 20 °C). The diffusion zone at the Ti/Cu interface, resulting from the extrusion process and the treatment after this process, has not been found.

**Figure 13:**
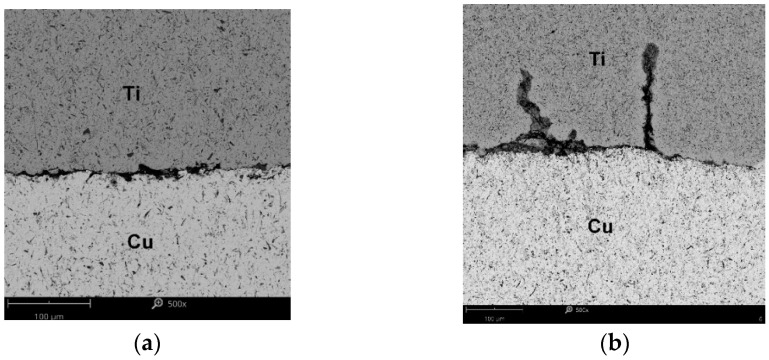
The contact zone of titanium and copper in the bimetal after annealing: (**a**) at *T* = 600 °C and *t* = 90 min, (**b**) at *T* = 800 °C and *t* = 60 min.

**Figure 14:**
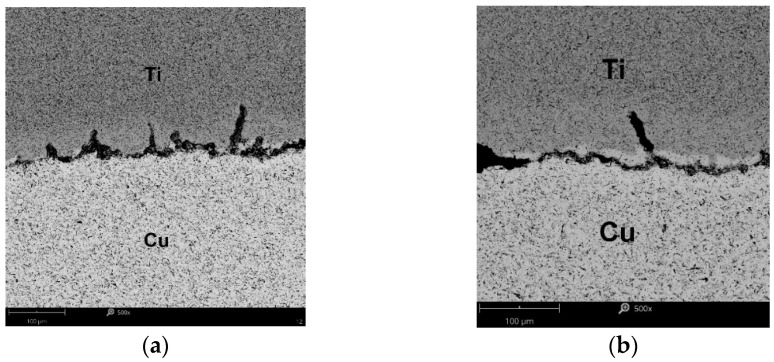
The contact zone of the titanium and copper layer in the bimetal after annealing at *T* = 900 °C and during: (**a**) *t* = 60 min, (**b**) *t* = 90 min.

Figure 13 and Figure 14 show the characteristic zones of the joining of Ti and Cu layers.

The first microcracks on the interface appeared after annealing the samples for 90 min at 600 °C (Figure 13a), which was not observed at the shorter annealing times. According to Matsuhita [16], this effect was found after 20 min and for *T* = 360 °C. A similar fact was also noted in [19,25] on the samples of Ti/Cu bimetals obtained with the explosive method and after post-process annealing at temperatures above 400 °C. At higher temperatures (700 °C and 800 °C), more cracks appeared, joining each other and displacing perpendicular to the lamination (Figure 13b). In addition, diffusion zones were formed where Ti and Cu adhered, the thickness of which increased significantly with the annealing time and was the highest at the temperature of 900 °C (Figure 14a). In samples after annealing at 900 °C and for *t* = 90 min, cracks within the copper layer were observed (Figure 14b).

The temperature of 900 °C in the Ti/Cu bimetal caused further degradation of the connection of the layers. As a result of the diffusion of copper into titanium, an increasingly thicker interfacial layer was formed. As reported by authors of the research works [19] and [12], in the vicinity of the temperature of 800–900 °C, the following phases occurred: TiCu; Ti_2_Cu; Ti_3_Cu_4_; TiCu_4_. With increasing temperature and holding time, the bond strength of the layers decreased because titanium (α – Ti) with an HCP structure became more easily diffusible compared to titanium (β – Ti) with a BCC structure. It affected the strength of the bimetal as a whole, as illustrated in Figure 7, showing the reduction in the tensile strength *R_m_* values under these conditions. The authors of the paper at [17] observed similar phenomena during the production of Ti/Cu bimetal by extrusion. A detailed analysis of the diffusion process at the titanium–copper interface was described in [41], considering the activation energy and purity of these metals. The method of identifying the intermetallic diffusion phases formed in the bimetal using the SEM technique is presented in the works [42,43]. In addition, the corrosion factors and the effect of heat treatment were taken into account there.

## 7. Conclusions

1. In the tested temperature range, the highest value of Young’s modulus *E* of the Ti/Cu bimetal occurred for T = 900 °C. It was 118 GPa for an annealing time of 90 min, while in the temperature range of 600–800 °C, its increase was insignificant and was in the range of 110–111 GPa. The annealing time had the most significant impact on the samples exposed to the temperature of 900 °C, characterized by an increase from 114 GPa to 118 GPa in the range of 30–90 min. Inverse relations occurred about the internal friction parameter *Q*^−1^ ranging in the temperature range of 600–700 °C. The highest value of the $Q^{-1}=1.26\cdot 10^{-3}$ parameter was recorded in the case of *t* = 90 min;
2. All samples of Ti/Cu subjected to the thermal factor showed several times decreased values of strength parameters concerning the virgin samples. The tensile strength Rm decreased from the level of 464.4 MPa to the value of 284.4 MPa with the temperature increase at T = 900 °C and t = 90 min. At the same time, the energy demand necessary for uniform plastic deformation (*L*_p_) of the samples increased by about ten times, from 9.1 MJ/m^3^ to 80.7 MJ/m^3^. At the temperature of 900 °C and for t = 90 minutes, the *L*_p_ value decreased by 58.0 MJ/m^3^ as the consequence of structural changes in the Ti/Cu bimetal. It was observed that even a 30-min overheating of the bimetal at 600 °C caused significant deterioration of the strength properties and the ability to work under load;
3. Increasing the holding time from 30 min to 90 min had the most substantial influence on the hardening factor *n* of samples annealed at 900 °C and caused its value to drop from 0.290 to 0.222. For the remaining temperatures in the range of 600–800 °C, the increase of the n coefficient was insignificant;
4. The heating of the Ti/Cu bimetal to the temperature of 900 °C caused, in the range of the tested annealing times, dangerous structural changes, causing destructive processes leading to the reconstruction of the internal structure, brittleness of the titanium layer, formation of gaps on the interface, and finally delamination.

## Figures and Tables

**Table 1:** Chemical composition of the titanium (Ti) and copper (Cu) layers [28].

**Titanium Layer (Ti) (%), Grade TA2**							
**Ti**	**Fe**	**C**	**N**	**H**	**0**	**others**	
99.6	0.002	0.003	0.002	0.0005	0.001	<0.4	
**Copper Layer (Cu) (%); Grade T2**							
**Cu + Ag**	**Bi**	**Sb**	**As**	**Fe**	**Pb**	**S**	**others**
99.9	0.03	0.002	0.004	0.005	0.004	0.005	0.05

**Table 2:** Basic elastic properties of Ti/Cu bimetal.

Temperatureof Annealing	Timeof Annealing	*E*	*χ_f_*	*Q* ^−1^
*T* (°C)	*t* (min)	(Gpa)	(Hz)	-
20	–	112.9	3493	8.65 × 10^−4^
600	30	110.1	3494	8.22 × 10^−4^
	60	110.5	3545	8.39 × 10^−4^
	90	109.7	3576	7.50 × 10^−4^
700	30	109.7	3534	1.07 × 10^−3^
	60	110.1	3542	9.82 × 10^−4^
	90	111.0	3568	1.07 × 10^−3^
800	30	111.3	3560	1.05 × 10^−3^
	60	110.9	3585	9.90 × 10^−4^
	90	111.7	3625	1.26 × 10^−3^
900	30	114.2	3537	1.00 × 10^−3^
	60	116.4	3591	1.04 × 10^−3^
	90	118.1	3623	1.18 × 10^−3^

**Table 3:** The values of the mechanical properties of Ti/Cu bimetal obtained from tensile tests.

Temperature of Annealing	Time of Annealing	*R_p_*_0.05_	*R_p_*_0.2_	*R_m_*	$\varepsilon_{u_{max}}$	*L_e_*	*L_p_*
*T* (°C)	*t* (min.)	(MPa)	(Mpa)	(MPa)	(mm/mm)	(MJ/m^3^)	(MJ/m^3^)
20		374.6	439.5	464.4	0.02	0.96	9.1
600	30	109.2	130.7	283.2	0.28	0.36	73.0
	60	97.7	122.7	280.2	0.27	0.36	69.1
	90	97.8	121.6	277.5	0.28	0.35	72.5
700	30	100.6	123.3	282.9	0.31	0.37	79.2
	60	92.6	115.7	270.3	0.31	0.33	76.9
	90	88.2	112.5	267.8	0.31	0.32	77.0
800	30	111.4	138.5	295.9	0.29	0.40	77.5
	60	113.5	138.2	295.7	0.29	0.39	78.4
	90	113.5	139.5	294.9	0.29	0.39	80.7
900	30	115.6	142.8	274.1	0.13	0.32	32.6
	60	129.8	166.4	287.5	0.09	0.36	23.8
	90	124.7	161.1	284.4	0.08	0.34	22.7

**Table 4:** Values of the hardening curve coefficients approximated by the Swift Equation (5).

Temperature of Annealing	Time of Annealing	*K*	*n*	$\varepsilon_{o}$
*T* (°C)	*t* (min)	(MPa)	-	(mm/mm)
20		520.4	0.022	5.19 × 10^-3^
600	30	535.7	0.278	1.74 × 10^-3^
	60	530.5	0.279	1.60 × 10^-3^
	90	525.1	0.278	1.76 × 10^-3^
700	30	519.5	0.260	9.59 × 10^-4^
	60	505.6	0.276	2.22 × 10^-3^
	90	503.1	0.281	2.72 × 10^-3^
800	30	542.6	0.262	3.56 × 10^-3^
	60	545.3	0.266	3.86 × 10^-3^
	90	548.9	0.276	4.70 × 10^-3^
900	30	565.2	0.290	6.59 × 10^-3^
	60	550.8	0.233	2.67 × 10^-3^
	90	532.6	0.222	9.78 × 10^-4^

## Data Availability

Not applicable.
